# Reliability and validity of the multidimensional impact of cancer risk assessment (MICRA) questionnaire: Japanese version

**DOI:** 10.1007/s10689-025-00496-2

**Published:** 2025-09-03

**Authors:** Tomoko Watanabe, Kaori Kimura, Minako Kakimoto, Yumie Hiraoka, Manami Matsukawa, Hiroko Nagahashi, Saki Horiguchi, Miwa Toshima, Chikako Tomozawa, Miki Aitani, Takeshi Kuwata, Teruhiko Yoshida, Makoto Hirata, Noriko Tanabe

**Affiliations:** 1 Department of Genetic Medicine and Services, National Cancer Center Hospital, 5-1-1 Tsukiji, Chuo-Ku, Tokyo 104-0045 Japan; 2 Department of Genetic Medicine and Services, National Cancer Center Hospital East, 6-5-1 Kashiwanoha, Kashiwa-Shi, Chiba 277-8577 Japan; 3 Department of Nursing, National Cancer Center Hospital, 5-1-1 Tsukiji, Chuo-Ku, Tokyo 104-0045 Japan; 4 Department of Clinical Genetics, Saitama Medical Center, Saitama Medical University, 1981 Kamoda, Kawagoeshi-Shi, Saitama 350-8550 Japan

**Keywords:** Hereditary breast and ovarian cancer, Multidimensional impact of cancer risk assessment (MICRA) questionnaire, *BRCA1*/*2* genetic testing, Psychological scale

## Abstract

This study aimed to develop and validate a Multidimensional Impact of Cancer Risk Assessment questionnaire–Japanese version (MICRA-J) as an assessment of the psychosocial impact of genetic testing. The MICRA was translated into Japanese using standardized translation procedures. The reliability and validity of the MICRA-J were evaluated in individuals who underwent *BRCA1*/*2* testing for hereditary breast and ovarian cancer diagnosis. The 72 respondents included patients with *BRCA1*/*2* pathogenic variants (n = 20), *BRCA1*/*2* negative (n = 35), variants of uncertain significance (VUS, n = 6), participants with cascade testing positive (n = 7), and participants with cascade testing negative (n = 4). Total MICRA-J scores were positively correlated with Hospital Anxiety and Depression Scale (HADS) scores and negatively correlated with the 36-item short-form version 2 acute (SF-36v2 acute) scores (*P* < 0.05). The MICRA-J showed good internal consistency coefficients (α > 0.70). Furthermore, high test–retest correlations were obtained when the 64 participants responded to the MICRA-J twice within a short period (Pearson’s correlation coefficient = 0.85). The MICRA-J Total score was higher in the groups with *BRCA1*/*2* pathogenic variant carriers than in the *BRCA1*/*2* negative group, whereas the HADS and SF36v2 acute did not differ significantly. These results suggest that the reliability and validity of the MICRA-J have been established. The MICRA-J, similar to the MICRA in other languages, is considered a useful tool to specifically measure the psychosocial impact of genetic testing.

**Supplementary Information:**

The online version contains supplementary material available at 10.1007/s10689-025-00496-2.

## Background

The number of individuals who undergo genetic testing and are diagnosed with cancer predisposition syndrome is increasing because advances in genetic analysis technology have led to the accumulation of evidence on cancer predisposition syndromes [1]. Moreover, the presence of variants in causative genes of cancer predisposition syndromes is sometimes used as a biomarker for treatment selection [2]. Since April 2020, *BRCA1*/*2* genetic testing has been covered by the national health insurance system in Japan for the diagnosis of hereditary breast and ovarian cancer (HBOC) in patients with breast cancer who meet specific criteria and in all with ovarian cancer [3]. HBOC practice guidelines have been developed worldwide, including in Japan, with detailed surveillance for early detection and the recommendation or discussion of prophylactic resection when HBOC is diagnosed [1, 3]. However, it is essential to understand the psychosocial impact of genetic testing on patients and their families when communicating genetic information. To establish a psychosocial support system in this process, an assessment scale for psychosocial impact, specific to genetic information, would be helpful.

In cancer predisposition syndromes, the Multidimensional Impact of Cancer Risk Assessment (MICRA) questionnaire has been developed in the USA as a scale to specifically measure the psychosocial impacts of genetic testing [4] because psychosocial distress related to the genetic testing of cancer predisposition syndromes cannot be fully evaluated using general psychometric tools [5]. The MICRA consists of 25 questions and has three subscales: “Positive experiences”, “Distress”, and “Uncertainty” [4]. The MICRA has been used in previous studies of individuals who underwent genetic testing for various cancers [6–14]. In those studies, the MICRA was translated into Portuguese and Norwegian [7, 10, 11]. However, a MICRA-Japanese version (MICRA-J) has not been established yet, and the reliability and validity of a MICRA-J must be assessed before implementing it to measure the psychosocial impact of genetic testing.

Thus, the current study aimed to develop a MICRA-J as an assessment tool to investigate the multidimensional impacts of genetic testing for cancer predisposition syndromes. The validity and reliability of the MICRA-J were examined through a questionnaire survey of participants who underwent *BRCA1*/*2* genetic testing. As a secondary objective, the results of the newly developed MICRA-J were also examined to reveal some psychosocial characteristics of individuals who have undergone *BRCA1*/*2* genetic testing.

## Methods

### Questionnaire translation and cultural adaptation

The MICRA-J was translated based on “A report of the International Society for Pharmacoeconomics and Outcomes Research (ISPOR) Task Force for translation and cultural adaptation” [15]. Permission to translate the MICRA was obtained from one of the authors (Dr. Lerman) and from the publisher (American Psychological Association, Inc.). In addition, we obtained permission from Dr. Cella and a license from the FACIT organization to translate the MICRA into Japanese. The English version of the MICRA was first translated by a professional translator, whose first language is Japanese. This translated version was then revised by a genetic counselor specializing in oncology, a native Japanese speaker who speaks English. Finally, a third person bilingual in Japanese and English with specialist skills in health psychology, interviews, and questionnaire translation made the final revisions. Back-translation was conducted by a Japanese–English bilingual speaker who was different from the translator. Another Japanese–English bilingual translator compared the back-translation of the MICRA-J with the original text to ensure that the translation was appropriate. The final version of the scale was completed after discussion and consensus among the translators.

Prior to the main study, a pilot study was conducted, which aimed to obtain feedback on the clarity and wording of the provisional MICRA-J from a public perspective. This pilot study was conducted between December 2021 and February 2022. Five individuals who had received genetic counseling and had undergone *BRCA1*/*2* genetic testing or cascade testing at the National Cancer Center Hospital (NCCH) participated in this pilot study. All participants were female, aged 30–69 years, two were carriers of *BRCA1*/*2* pathogenic variants (one of whom had a variant proven with cascade testing), and three were *BRCA1*/*2* negative with a history of cancer. Since most of them (n = 4) indicated that the time point asked for in their responses was unclear, “in the past week” was emphasized. In addition, a few words of the MICRA-J were changed while taking into consideration whether the original text and the back-translation would not diverge, and the wording of the MICRA-J was finalized.

### Participants

This study was approved by the NCC Research Ethics Review Committee (2021–284). In both the pilot and main studies, written informed consent was obtained from all participants. Participants were recruited from among individuals who had undergone *BRCA1*/*2* genetic testing at the NCCH or NCCH East, regardless of the presence or absence of a history of cancer. The participants were recruited during outpatient visits between June 2022 and November 2023. The time of the outpatient visits was not necessarily limited to the appointment on which the genetic test results were disclosed. This study included individuals within approximately three years of disclosure of the results because the primary objective of this study was to evaluate the reliability and validity of the MICRA-J. Reliability was assessed by determining the similarity of responses from the same individual in the first and second rounds of the survey. The psychosocial effects of a genetic diagnosis are considered to be stronger during the immediate post-diagnosis period [16]. Immediately after diagnosis, the psychological impact is significant, and it is predicted that this impact will subside temporarily over time. Therefore, participants were asked to complete the questionnaire one month after diagnosis or later.

The sample size was set based on the previous reports in which the Portuguese translation of the MICRA [10] had been validated with 96 individuals (all variant carriers, 54 developed related tumors of *SHDx* mutations), the Norwegian translation of the MICRA included 32 ovarian cancer *BRCA1*/*2* variant carriers [7], and the original MICRA included *BRCA1*/*2* negative and true negative cases [4].

For the analysis of psychosocial characteristics, participants were divided into five groups. Participants with a history of cancer were divided into three groups based on their genetic testing results: with *BRCA1*/*2* pathogenic or likely pathogenic variants (Group 1: positive), without reportable *BRCA1*/*2* variants (i.e., neither pathogenic/likely pathogenic variants, variants of uncertain significance (VUS), nor other special comments) (Group 2: negative), or with VUS (Group 3: VUS). Participants without a history of cancer were divided into two groups based on the results of cascade testing for the presence of a pathogenic variant detected in their family: with (Group 4: cascade-positive) and without (Group 5: cascade-negative) the variant. To examine the effects of a history of cancer, participants were also divided into two groups: those with and without a history of cancer.

### Procedure

Participants were asked to complete two sets of questionnaires. They could choose between web- or paper-based questionnaires as their response method. While requesting responses to the first set of MICRA-J, we also distributed a second set of MICRA-J (a two-dimensional barcode providing access to a response website for those who chose to respond online) and requested responses approximately one week later. The first set of questionnaires consisted of participant demographics, the MICRA-J, Hospital Anxiety and Depression Scale (HADS), and 36-item short-form (SF-36) version 2 acute. The second set comprised only the MICRA-J. Details of each scale are described below in the “Questionnaires” section. The attributes of the study participants were covered using five questions (age, marital status, education, income, and employment). To examine the test–retest reliability of the MICRA-J, participants were asked to respond to the first and second questionnaires more than one week apart. Since the purpose of the test re-test was to confirm reproducibility, a range in response times was acceptable.

### Questionnaires

#### Multidimensional impact of cancer risk assessment (MICRA) questionnaire

The MICRA was developed by Cella and colleagues to specifically measure the psychosocial impacts of genetic testing [4]. The MICRA consists of 25 items, including 21 questions to be completed by all respondents, two questions to be answered by individuals with children, and two questions to be answered by individuals with a history of cancer [4]. Each question is answered on a four-point scale of “never,” “rarely,” “sometimes,” and “often”; the MICRA Total score was the sum of the scores for the 21 questions and ranges from 0 to 105, with higher scores indicating a stronger psychosocial impact. The study identified three subscales [4]: Distress (0–30 points), Uncertainty (0–45 points), and Positive experiences (0–20 points). Items #5, #6, #18, and #19 of the Positive experience subscale, as well as item #13, were reversed when included in the total score. Two items not included in the three subscales (items #13 and #21) were included in the total score. The scoring of the MICRA-J aligns with the MICRA scoring.

#### Hospital anxiety and depression scale (HADS)

The HADS was developed as a self-assessment scale to detect anxiety and depression in patients with physical symptoms [17]. Covering a total of 14 items, the subscales Anxiety (7 items) and Depression (7 items) were measured on a 4-point Likert scale (0–3). Each subscale score ranged from 0 to 21 points. Cutoff values were determined for each item as “no anxiety or depression” (0–7 points), “suspicious” (8–10 points), and “anxiety or depression” (> 10 points). The HADS has been validated for scale validity and reliability in the Japanese population [18].

#### The 36-item short-form (SF-36) version 2 acute

The SF-36 is a widely used quality-of-life scale that measures overall health status but not specific diseases or symptoms [19]. The validity of the Japanese translation of the SF-36 has already been confirmed [20]. In line with the MICRA questionnaire regarding conditions in the past week, the SF-36 version 2 acute (SF-36v2 acute) scale was used to measure mental health items (Vitality, Social functioning, Role-emotional, and Mental health) and General health perception. For the Japanese version of the SF-36, norm-based scoring was calculated using Japanese standard values from the 2017 national survey (converting the national standard value to 50 points and its SD to 10 points) [21]. As standard values for the SF-36v2 acute in Japan are unavailable, the data were interpreted using scores calculated with reference to SF-36 standard values, with the caveat that direct comparisons cannot be made.

### Data analyses

First, Fisher’s exact test was conducted to check for differences in the proportions of age, cancer status, and genetic test results between respondents and non-respondents in the study participants. Thereafter, the Kruskal–Wallis or Mann–Whitney *U* test was conducted to check for differences in the mean age at diagnosis, age at survey, time from disclosure of test results to survey, and interval between questionnaires among Groups 1–3 or 4–5. Fisher’s exact test was conducted to check for differences in the proportions of respondents’ demographics among the groups.

Prior to analyzing the scores for each questionnaire, estimates were assigned to missing values using the Expectation Maximization (EM) Algorithm (SPSS Missing values). Mean scores and SDs were calculated for each group for the MICRA-J Total score and its three subscale scores, HADS scores, and SF-36v2 acute scores. Kruskal–Wallis and Mann–Whitney *U* tests were also performed to check for differences in the mean scores among Groups 1–3 and 4–5, respectively. When performing these tests, individuals with and without a history of cancer were tested separately. Significance values were adjusted by the Bonferroni correction for multiple tests in post-hoc analyses when the Kruskal–Wallis tests indicated significant results. Correlation coefficients among the MICRA-J, HADS, and SF-36v2 acute scores were calculated to examine the validity of the MICRA-J scale. Internal consistency was also assessed by calculating Cronbach’s alpha coefficients for all MICRA-J responses. Reliability was then examined by calculating Pearson’s correlation coefficients for the MICRA-J Total score using the scores of individuals who responded to both the first and second questionnaires. Correlation coefficients (absolute values of r) of 0.2–0.39, 0.4–0.69, and 0.7–1.0 were considered to indicate weak, moderate, and strong correlations, respectively.

In addition, to determine how cancer status and genetic test results for participants affected psychosocial aspects that can be measured by the MICRA-J, each item of the MICRA-J was dichotomized into infrequent (“never” and “rarely”) and frequent (“sometimes” and “often”). To examine the factors associated with higher MICRA-J scores, univariate linear regression analysis was performed using the MICRA-J Total scores as the dependent variable and other factors as independent variables, and multivariate linear regression analysis was performed for items that were significant in these univariate analyses, accounting for multicollinearity. Data were analyzed using IBM SPSS Statistics version 29. Statistical significance was defined as *p* < 0.05 for all analyses. This study follows the STROBE guidelines for reporting observational studies.

## Results

### Participants

**Fig. 1:**
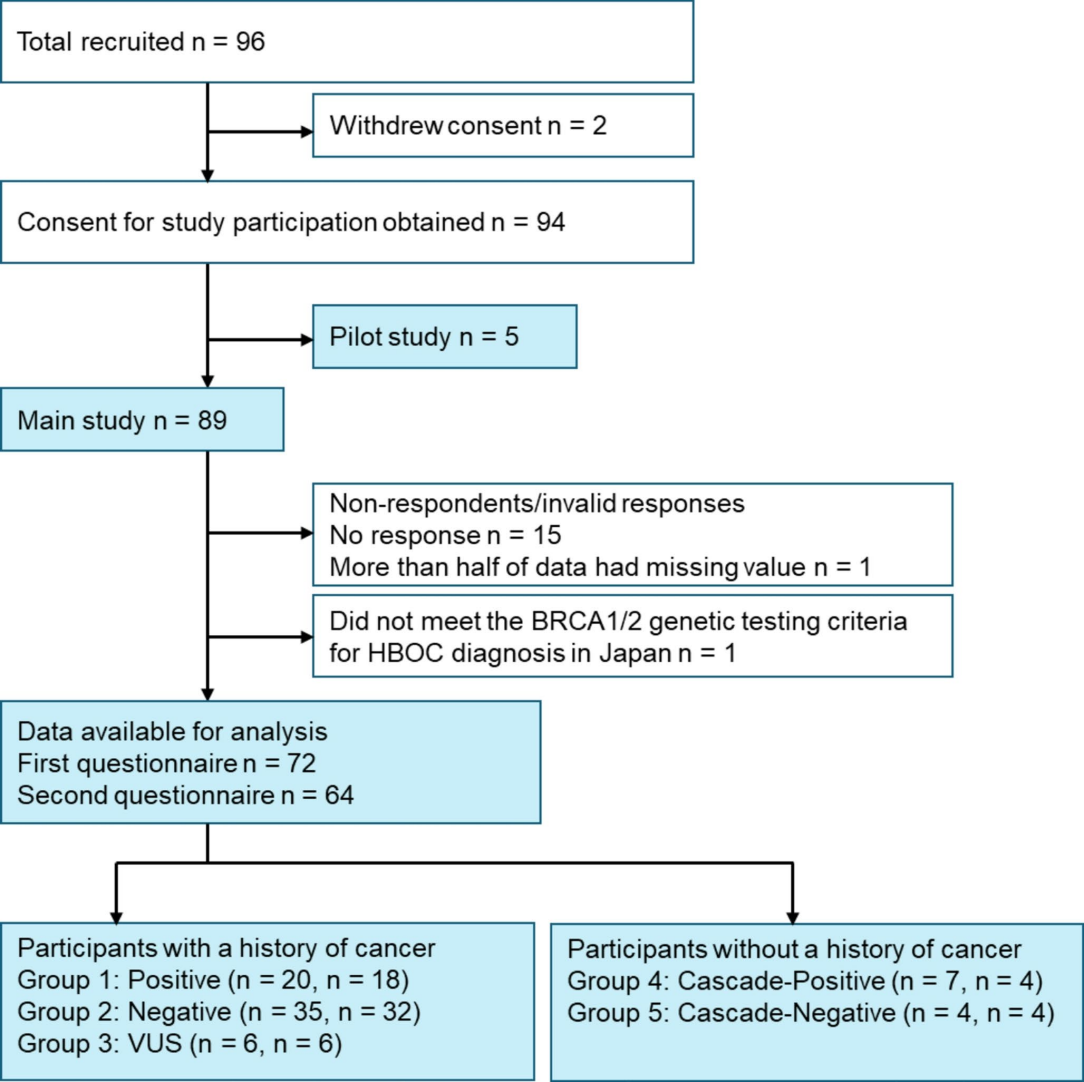
A flow diagram of this study

Consent for study participation was obtained from 94 individuals, excluding two who initially agreed to participate in the study but withdrew their consent before answering the questionnaires. Five participants were asked to provide their opinion on the MICRA-J as a pilot study, and 89 were asked to answer a questionnaire containing the MICRA-J as the main study. The first set of questionnaires was completed by 74 of the 89 participants (including one man; response rate: 83.1%). The second set of questionnaires was completed by 66 of the 89 participants (response rate: 74.2%); one participant was excluded from this analysis because more than half of the data had missing values. For three missing values, one item from each of the three participants (3 of 138 valid responses; 2.2%), the estimates were assigned as described in the Methods section. The three items with missing values included one item each from the MICRA-J and HADS in the first questionnaire and one item from the MICRA-J in the second questionnaire. Among respondents with a history of cancer, all but one had a history of cancer and met the *BRCA1*/*2* genetic testing criteria for HBOC diagnosis [3]. In order to reduce heterogeneity among the patients with cancer, the analysis was conducted after excluding data for one patient who did not meet the *BRCA1*/*2* genetic testing criteria. Finally, 72 of 89 (80.9%) valid responses were received for the first questionnaire and 64 of 89 (71.9%) for the second questionnaire. The above-mentioned information is summarized as a flow diagram (Fig. 1). Age, history of cancer, and genetic test results did not significantly differ between respondents (n = 72) and non-respondents/non-valid respondents (n = 16; Table S1 in Online Resource 1).

Among the 72 respondents, the mean time from the disclosure of the genetic test results to the first questionnaire response was 432 days (SD: 344, range 2–1149). We examined whether the proportions of individual attributes among groups with a history of cancer (Group 1 vs. Group 2 vs. Group 3) and between groups without a history of cancer (Group 4: cascade-positive vs. Group 5: cascade-negative) differed. The time from the disclosure of the genetic test results to the first questionnaire response was significantly longer in Group 4: cascade-positive than in Group 5: cascade-negative (Table 1). Other attributes related to HBOC diagnosis are shown in Table S2 in Online Resource 1.

**Table 1:** Characteristics of the study groups

	Group 1 positive		Group 2 negative		Group 3 VUS		*P*-value	Group 4 cascade-positive		Group 5 cascade-negative		*P*-value	Total	
	n		n		n			n		n			n	
First questionnaire	20		35		6			7		4			72	
Second questionnaire	18		32		6			4		4			64	
	Mean	SD	Mean	SD	Mean	SD		Mean	SD	Mean	SD		Mean	SD
Age at first diagnosis of HBOC-related cancer (years)	46.8	13.4	47.1	10.7	47.5	12.5	0.84^a^	NA		NA			47.1	11.6
Age when surveyed (showing the age at which consent was obtained) (years)	50.8	13.8	51.7	10.8	47.7	11.9	0.47^a^	43.3	11.8	48.0	5.6	0.41^b^	50.1	11.7
Time from disclosure of test results to survey (days)	407	335	506	355	171	144	0.07^a^	568	318	66.3	48.4	0.01^b^	432	344
Interval between questionnaires (days)	21.7	35.6	12.5	14.2	12.3	6.2	0.63^a^	13.5	4.2	7.8	7.0	0.20^b^	14.8	21.6
	n	%	n	%	n	%		n	%	n	%		n	%
*Age (years)*														
< 50	9	45%	13	37%	5	83%	0.12^c^	5	71%	2	50%	0.58^c^	34	47%
≥ 50	11	55%	22	63%	1	17%		2	29%	2	50%		38	53%
*Marital status*														
Married	11	55%	20	57%	4	67%	1.00^c^	3	43%	4	100%	0.20^c^	42	58%
Not married	9	45%	14	40%	2	33%		3	43%	0	0%		28	39%
Do not want to answer	0	0%	1	2.9%	0	0%		1	14%	0	0%		2	2.8%
*Education level*														
College graduate	8	40%	16	46%	2	33%	0.80^c^	5	71%	3	75%	1.00^c^	34	47%
Some college or less	12	60%	19	54%	4	67%		2	29%	1	25%		38	53%
*Income level*														
≥ 5 million JPY	7	35%	15	43%	2	33%	0.78^c^	5	71%	4	100%	0.49^c^	33	46%
< 5 million JPY	9	45%	15	43%	4	67%		2	29%	0	0%		30	42%
Do not want to answer	4	20%	5	14%	0	0%		0	0%	0	0%		9	13%
*Employment status*														
Employed	12	60%	25	71%	5	83%	0.44^c^	5	71%	4	100%	0.49^c^	51	71%
Not employed	8	40%	8	23%	1	17%		2	29%	0	0%		19	26%
Do not want to answer	0	0%	2	5.7%	0	0%		0	0%	0	0%		2	2.8%
*Children*														
Yes	11	55%	17	49%	4	67%	0.80^c^	4	57%	4	100%	0.24^c^	40	56%
No	9	45%	18	51%	2	33%		3	43%	0	0%		32	44%

^a^Kruskal–Wallis test, ^b^Mann–Whitney *U* test, ^c^Fisher’s exact test. The test excluded those who responded with “Do not want to answer.”

NA: not applicable

### Pearson’s correlation coefficients between scale items

The MICRA-J Total score was positively correlated with each subscale of the MICRA-J (Positive experiences [reversal items], Distress, and Uncertainty) and the Anxiety and Depression subscales of the HADS scale (Table 2). In contrast, the MICRA-J Total scores were negatively correlated with each item of the SF-36v2 acute (i.e., General health perception, Vitality, Social functioning, Role-emotional, and Mental health; Table 2). The correlation was strong for the MICRA-J subscales and weak to moderate for the subscales of other scales.

**Table 2:** Pearson’s correlation coefficients between scale items

	MICRA positive experiences	MICRA distress	MICRA uncertainty	HADS anxiety	HADS depression	GH (N)	VT (N)	SF (N)	RE (N)	MH (N)
MICRA total score	0.71**	0.84**	0.83**	0.53**	0.34**	− 0.45**	− 0.36**	− 0.38**	− 0.23*	− 0.39**
MICRA positive experiences	–	0.45**	0.35**	0.14	0.21	− 0.26*	− 0.27*	− 0.35**	− 0.16	− 0.23*
MICRA distress		–	0.57**	0.51**	0.23	− 0.27*	− 0.20	− 0.28*	− 0.14	− 0.30*
MICRA uncertainty			–	0.59**	0.35**	− 0.52**	− 0.36**	− 0.26*	− 0.22	− 0.37**
HADS anxiety				–	0.64**	− 0.58**	− 0.53**	− 0.42**	− 0.43**	− 0.75**
HADS depression					–	− 0.61**	− 0.73**	− 0.53**	− 0.62**	− 0.74**
GH (N)						–	0.72**	0.45**	0.40**	0.61**
VT (N)							–	0.47**	0.55**	0.72**
SF (N)								–	0.50**	0.53**
RE (N)									–	0.54**

^*^ *P* < 0.05, ** *P* < 0.01

*GH*: General health perception, *VT*: Vitality, *SF*: Social functioning, *RE*: Role-emotional, *MH*: Mental health, *N*: normalized SF-36v2 acute score

### Internal consistency coefficients of the MICRA-J scale

Cronbach’s coefficient α for the MICRA-J Total score and its three subscales were as follows (the values reported by Cella et al. [4] are shown in parentheses): MICRA-J Total score α = 0.87 (0.77), Distress α = 0.86 (0.86), Uncertainty α = 0.72 (0.77), and Positive experiences α = 0.72 (0.75).

### Reliability of the MICRA-J scale

Among the 64 participants who responded to the first and second questionnaires, Pearson’s correlation coefficient between the first and second MICRA-J Total scores was 0.85 (*p* < 0.01). The mean MICRA-J Total score of the 72 respondents in the first set of questionnaires was 22.6 (SD: 14.1; Table 3). The MICRA-J Total score of the respondents in the second questionnaire was 23.0 (SD: 14.4). The mean time between the first and second responses in participants who responded to both the first and second questionnaires was 14.8 days (standard deviation [SD]: 21.6, range 0–133).

**Table 3:** Mean scores of the first questionnaire by group

Scales	No. of items	Group 1 positive		Group 2 negative		Group 3 VUS			Group 4 cascade-positive		Group 5 cascade-negative			Total	
		n = 20		n = 35		n = 6			n = 7		n = 4			n = 72	
		Mean	SD	Mean	SD	Mean	SD	*p* value^a^	Mean	SD	Mean	SD	*P*-value^b^	Mean	SD
MICRA total	21	32.2	15.2	16.1	10.6	31.0	15.3	< 0.01	25.7	7.1	12.8	9.1	0.04	22.6	14.1
MICRA positive experiences	4	8.4	4.1	3.0	3.8	9.0	3.9	< 0.01	10.9	6.1	1.5	1.9	0.01	5.7	5.1
MICRA distress	6	10.2	6.8	2.5	2.7	7.8	8.0	< 0.01	5.7	2.8	2.8	4.9	0.16	5.4	5.8
MICRA uncertainty	9	12.5	7.0	9.8	6.0	12.2	6.4	0.43	9.0	5.5	7.0	4.7	0.79	10.5	6.3
HADS anxiety	7	6.7	4.2	5.7	4.0	5.3	2.9	0.72	4.1	3.8	3.8	3.9	1.00	5.7	3.9
HADS depression	7	5.3	2.9	4.7	4.4	4.3	3.3	0.54	4.6	6.3	3.8	4.1	1.00	4.8	4.1
GH (N)	5	44.0	10.2	45.8	10.1	46.4	2.8	0.46	50.5	15.9	54.5	5.6	0.65	46.3	10.3
VT (N)	4	46.0	9.1	48.9	11.0	46.3	7.5	0.47	51.1	15.0	53.6	6.7	1.00	48.4	10.5
SF (N)	2	44.5	11.6	48.5	10.7	37.1	16.9	0.16	48.9	16.5	50.7	5.4	0.53	46.6	12.1
RE (N)	3	44.5	10.4	43.7	10.1	38.2	14.1	0.57	42.5	18.0	50.3	7.7	0.65	43.7	11.3
MH (N)	5	50.1	8.4	52.1	9.7	51.6	7.6	0.69	53.5	15.0	53.9	8.6	0.79	51.8	9.6

^a^Kruskal–Wallis test, ^b^Mann–Whitney *U* test, *SD*: standard deviation, *GH*: General health perception, *VT*: Vitality, *SF*: Social functioning, *RE*: Role-emotional, *MH*: Mental health, *N*: normalized SF-36v2 acute score

### Comparison of the scores from the MICRA-J and other scales among study groups

The MICRA-J Total scores differed significantly among the three groups of individuals with a history of cancer (Group 1: positive, Group 2: negative, and Group 3: VUS) (Table 3). Post-hoc analyses revealed that the mean MICRA-J Total scores were significantly higher in the positive group than the negative group, as were the scores for the MICRA-J subscale Distress. The mean scores for the MICRA-J subscale Positive experiences differed significantly among the positive–negative groups and the VUS-negative groups, respectively. In contrast, the MICRA-J subscale Uncertainty, HADS, and SF-36v2 acute showed no significant differences among the three groups. Between the two groups without a history of cancer undergoing cascade testing (Group 4: cascade-positive and Group 5: cascade-negative), the MICRA-J Total and the MICRA-J subscale Positive experiences score was significantly higher in the positive group than in the negative group. The MICRA-J subscales Distress and Uncertainty, HADS, and SF-36v2 acute did not differ significantly between the two groups.

When the two groups were divided into those with and without a history of cancer, the General health perception score on the SF-36v2 acute scale was significantly lower in the group with a history of cancer than in the group without a history of cancer. However, no significant differences were found for the other scales, including the MICRA-J (Table S3 in Online Resource 1).

### Feasibility of the MICRA-J

Each MICRA-J item was dichotomized into infrequent (“never” and “rarely”) and frequent (“sometimes” and “often”; Table 4). When dichotomized, either of these two categories was less frequent than 20% in 12 of 21 items. Item #9 “Worrying about my risk of getting cancer (or getting cancer again if you have ever been diagnosed with cancer)” and item #13 “Understanding clearly my choices for cancer prevention or early detection” were answered by more than 80% of the respondents as “sometimes” or “often.” Notably, approximately 84% of the respondents with a history of cancer answered item #9 with “sometimes” or “often” despite negative genetic test results. However, no respondents (0/72; 0%) answered item #21 “Feeling regret about getting my test results” with “sometimes” or “often.”

**Table 4:** Frequency distributions of all MICRA items

		Group 1 positive		Group 2 negative		Group 3 VUS		Group 4 cascade-positive		Group 5 cascade-negative		Total	
		n = 20		n = 35		n = 6		n = 7		n = 4		n = 72	
Item		n^a^	%	n^a^	%	n^a^	%	n^a^	%	n^a^	%	n^a^	%
1	Feeling upset about my test result	7	35	1	2.9	2	33	2	29	1	25	13	18
2	Feeling sad about my test result	10	50	0	0	2	33	2	29	0	0	14	19
3	Feeling anxious or nervous about my test result	10	50	3	8.6	2	33	3	43	0	0	18	25
4	Feeling guilty about my test result	6	30	0	0	2	33	1	14	1	25	10	14
5	Feeling relieved about my test result	5	25	30	86	1	17	1	14	4	100	41	57
6	Feeling happy about my test result	6	30	34	97	1	17	2	29	4	100	47	65
7	Feeling a loss of control	6	30	8	23	2	33	0	0	1	25	17	24
8	Having problems enjoying life because of my test result	7	35	0	0	0	0	0	0	0	0	7	10
9	Worrying about my risk of getting cancer [or getting cancer again if you have ever been diagnosed with cancer]	16	80	30	86	5	83	5	71	3	75	59	82
10	Being uncertain about what my test result means about my cancer risk	4	20	6	17	3	50	0	0	0	0	13	18
11	Being uncertain about what my test result means for my child(ren) and/or family’s cancer risk	4	20	2	5.7	2	33	0	0	0	0	8	11
12	Having difficulty making decisions about cancer screening or prevention (e.g., having preventive surgery or getting medical tests done) ^b^	2	10	2	5.9	0	0	0	0	1	25	5	7.0
13	Understanding clearly my choices for cancer prevention or early detection	18	90	32	91	3	50	7	100	3	75	63	88
14	Feeling frustrated that there are no definite cancer prevention guidelines for me	5	25	9	26	1	17	1	14	0	0	16	22
15	Thinking about my test results has affected my work or family life	5	25	6	17	1	17	2	29	1	25	15	21
16	Feeling concerned about how my test results will affect my insurance status	7	35	7	20	1	17	1	14	1	25	17	24
17	Having difficulty talking about my test results with family members	3	15	4	11	2	33	1	14	1	25	11	15
18	Feeling that my family has been supportive during the genetic counseling and testing process	16	80	26	74	3	50	4	57	4	100	53	74
19	Feeling satisfied with family communication about my genetic test result	13	65	30	86	4	67	4	57	4	100	55	76
20	Worrying that the genetic counseling and testing process has brought about conflict within my family	6	30	2	5.7	0	0	0	0	0	0	8	11
21	Feeling regret about getting my test results	0	0	0	0	0	0	0	0	0	0	0	0

	Children												
		n = 11		n = 17		n = 4		n = 4		n = 4		n = 40	
		n^a^		n^a^		n^a^		n^a^		n^a^		n^a^	
22	Worrying about the possibility of my children getting cancer	11	100	17	100	4	100	3	75	1	25	36	90
23	Feeling guilty about possibly passing on the disease risk to my child(ren)	10	91	10	59	3	75	3	75	1	25	27	68

	History of cancer								
		n = 20		n = 35		n = 6		n = 61	
		n^a^		n^a^		n^a^		n^a^	
24	Feeling that the genetic test result has made it harder to cope with my cancer	10	50	15	43	5	83	30	49
25	Feeling that the genetic test result has made it easier to cope with my cancer	11	55	19	54	0	0	30	49

^a^Number of respondents who answered “sometimes” or “often.” ^b^ Calculated with n = 34 or n = 71 excluding missing value n = 1 (“1” in EM estimation)

*VUS*: variants of uncertain significance

Of the 40 individuals who answered that they have children, more than 90% (36/40) responded with “sometimes” or “often” to item #22 “Worrying about the possibility of my children getting cancer,” irrespective of the genetic results. More than half (27/40; 68%) also responded “sometimes” or “often” to item #23 “Feeling guilty about possibly passing on the disease risk to my child(ren).”

### Regression analysis

Univariate regression analysis showed that HADS scores (Anxiety and Depression), SF-36v2 acute scores (General health perception, Vitality, Social functioning, and Mental health), risk-reducing mastectomy, risk-reducing salpingo-oophorectomy, and the status of *BRCA1*/*2* variant carriers were significantly associated with the MICRA-J Total score (Table 5). Subsequently, multivariate linear regression was performed for items that were significant in the univariate regression analysis except for the variable genetic test result “negative vs others” due to multicollinearity. The MICRA-J Total score was significantly associated with the status of *BRCA1*/*2* pathogenic variant carriers and the HADS Anxiety score (Table 5).

**Table 5:** Linear regression analysis of MICRA Total score and independent variables

Variables	Univariate			Multivariate		
	B	β	*p*	B	β	*p*
Age at first diagnosis of HBOC-related cancer, n = 61	− 0.18	− 0.15	0.26			
Age when surveyed (showing the age at which consent was obtained)	− 0.18	− 0.15	0.22			
Time from disclosure of test result to survey	0.003	0.07	0.56			
HADS anxiety	1.90	0.53	< 0.01	1.69	0.47	< 0.01
HADS depression	1.19	0.34	< 0.01	− 0.42	− 0.12	0.45
GH (N)	− 0.61	− 0.45	< 0.01	− 0.32	− 0.23	0.10
VT (N)	− 0.48	− 0.36	< 0.01	− 0.02	− 0.01	0.94
SF (N)	− 0.44	− 0.38	< 0.01	− 0.22	− 0.19	0.10
RE (N)	− 0.29	− 0.23	0.05			
MH (N)	− 0.58	− 0.39	< 0.01	0.21	0.14	0.41
*BRCA1*/*2* variant status: positive	12.7	0.44	< 0.01	11.1	0.38	< 0.01
*BRCA1*/*2* variant status: VUS	9.20	0.18	0.13			
*BRCA1*/*2* variant status: negative	–14.8	–0.53	< 0.01			
Own cancer diagnosis	1.85	0.05	0.69			
Marital status: married, n = 70^a^	1.71	0.06	0.62			
Education: college graduate	− 0.58	− 0.02	0.86			
Income: ≥ 5 million JPY, n = 63^a^	0.78	0.03	0.83			
Employment status: employed, n = 70^a^	− 5.53	− 0.17	0.15			
Having children	1.53	0.05	0.65			
Early onset: 45 years old or younger	2.40	0.08	0.49			
TN: 60 years old or younger, n = 71^b^	6.86	0.11	0.35			
Two or more primary cancers	− 3.14	− 0.09	0.47			
Ovarian/fallopian tube/peritoneal cancer	8.11	0.16	0.18			
Breast cancer within TDR	− 0.90	− 0.03	0.80			
Ovarian cancer within TDR	0.37	0.01	0.94			
Pancreatic cancer within TDR	3.71	0.12	0.32			
Multiple cancers	− 3.56	− 0.11	0.34			
RRM	12.7	0.25	0.03	3.05	0.06	0.59
RRSO	15.7	0.29	0.02	− 0.71	− 0.01	0.91

^a^Those who responded with “Do not want to answer” were excluded. ^b^A case of unknown was excluded

*HBOC*: hereditary breast and ovarian cancer, *GH*: General health perception, *VT*: Vitality, *SF*: Social functioning, *RE*: Role-emotional, *MH*: Mental health, *N*: normalized SF-36v2 acute score, *TN*: triple-negative breast cancer, *TDR*: third-degree relatives, *RRM*: risk-reducing mastectomy, *RRSO*: risk-reducing salpingo-oophorectomy

Dummy variable: YES = 1, NO = 0

## Discussion

The total scores of the MICRA-J were positively correlated with the HADS scores and negatively correlated with the SF-36v2 acute scores, indicating that the psychosocial impact measured by the MICRA-J was correlated with general psychological distress. These results indicate that the validity of the MICRA-J was confirmed. Furthermore, the MICRA-J showed a significant difference in psychosocial impact between the positive genetic variant group and the negative genetic variant groups, whereas the HADS and SF-36v2 acute scales were unable to do so. Therefore, the MICRA-J, similar to the MICRA in other languages, is a specific measurement tool to assess the psychosocial impact of genetic testing. Furthermore, high test–retest correlations were obtained when the same participants responded twice within a short period. When comparing the results of this study with those of previous studies in individuals who had undergone *BRCA1*/*2* genetic testing, the mean total score of the MICRA-J for each group was within the approximate ranges reported previously [4, 7]. Thus, the reliability and validity of the MICRA-J were confirmed.

As a study population, no significant differences were detected for the attributes of respondents and non-respondents or among groups of respondents (Table 1, Table S1 in Online Resource 1). However, one reason for the significant difference in the time from the disclosure of the genetic test results to the first questionnaire response might be that Group 5 cascade-negative was asked to participate in the study shortly after the disclosure of the test results because no further hospital visits were scheduled, whereas Group 4 cascade-positive was asked to participate in the study at the time of surveillance or follow-up.

The MICRA-J score showed a significant difference in the mean values between the *BRCA1*/*2*-positive group and the *BRCA1*/*2*-negative group, whereas no significant differences in the mean HADS or SF-36v2 acute scores were found. In addition, the results of the regression analysis showed that the MICRA-J Total score was related to two factors: a positive genetic test result and the HADS Anxiety score. The mean MICRA-J Total score for all *BRCA1*/*2*-positive individuals in this study was 31 points. This score is almost the same as the cutoff value of 32 points used in a previous study [7] to define high MICRA Total scores. Those who were *BRCA1*/*2*-positive and had a HADS anxiety score ≥ 8 exceeded 31 points (8/9, 89%). However, even individuals who were *BRCA1*/*2*-positive and had HADS Anxiety scores < 8, 22% (4/18) had MICRA Total scores higher than 31 points. Thus, these results are consistent with previous reports that the psychosocial impact specific to genetic testing tends to be difficult to capture using only scales that measure general psychological distress [4, 7]. In addition, the presence or absence of a history of cancer was the only statistically significant factor in the General health perception score of the SF-36v2 acute scale, that is, the MICRA-J scores were not significantly different. This result also supports a previous finding [4] that the mean MICRA Total scores do not significantly differ between individuals with and without a history of cancer, also consistent with other previous results [11] showing that affected individuals with pathogenic variants report lower overall quality of life than non-affected carriers in hereditary paraganglioma-pheochromocytoma syndrome. Thus, the MICRA-J is a tool that can specifically measure the psychosocial impact of genetic testing.

Previous reports suggest that 20–30% of patients with breast or ovarian cancer have psychological scale scores indicative of anxiety [22, 23]. Although the HADS Anxiety scale does not capture all cases with high MICRA-J scores, in clinical practice, psychological support is considered particularly important for patients with high anxiety levels, not only in the treatment of breast and ovarian cancer but also before and after genetic testing.

The mean MICRA-J Total scores of the *BRCA1*/*2*-positive groups in this study (32.2 for Group 1 and 25.7 for Group 4) were within the approximate range of 27.5 for affected and unaffected women with *BRCA1*/*2* pathogenic variants in the original MICRA report [4] and 38.6 in a previous study of patients with ovarian cancer and *BRCA1*/*2* pathogenic variants [7]. The mean scores of the *BRCA1*/*2*-negative groups (16.1 for Group 2 and 12.8 for Group 5) were also similar to those from the original report on the MICRA (12.7–15.1) [4] and a previous study of patients with ovarian cancer (14.9–17.1) [7]. Compared with the findings of other studies [13, 14], the mean MICRA-J Total score of the VUS group in the present study was 31.0, which was relatively high. Half (3/6) of the respondents answered item #13 “Understanding clearly my choices for cancer prevention or early detection,” with “often/sometimes.” In previous reports, the MICRA Total scores for the VUS group, calculated on a 95-point scale excluding two items from the MICRA, were 12.4 for participants with a history of cancer [13] and 17.4 for participants undergoing multigene panel testing [14]. The majority of VUS cases are benign polymorphisms [24]. Although some of these may become pathogenic variants in the future, they were not clinically actionable in terms of genetic counseling at the time of disclosure. The higher MICRA-J Total scores in the VUS group may reflect an understanding of uncertainty. Previous studies have shown that the distress, depression, and anxiety of VUS cases are inconsistent [25, 26]. Moreover, factual recalls of the test results have been reported, which incorrectly understood VUS as the result of a pathogenic variant [26]. However, the VUS group only comprised six participants. Thus, further accumulation of cases is necessary to elucidate the trend of higher MICRA-J scores in the VUS group.

None of the participants in this study responded “often” or “sometimes” to item #21 “Feeling regret about getting my test results” (0/72; 0%), regardless of the genetic testing results. However, the majority of participants were worried about their risk of getting cancer or getting cancer again if they had ever been diagnosed with cancer (59/72, 82%) or the possibility of their children getting cancer (36/40, 90%). These findings regarding low levels of regret [6, 8] and high concern about the risk of cancer [11, 12] are similar to those of previous studies. The possibility remains that the respondents interpreted “risk of getting cancer” as “risk of recurrence” when answering the questionnaire. However, these findings suggest that despite not regretting the choice to undergo genetic testing, anxiety about the risk of developing cancer remains, regardless of the results of the genetic test.

We also considered the unique situation in Japan and the adaptation of the MICRA items. Since Japan has a national health insurance system and genetic testing and subsequent surveillance are covered by this insurance when certain criteria are met, we had concerns about the need for item #16 “Feeling concerned about how my test results will affect my insurance status.” However, 24% (17/72) of the respondents answered with “often” or “sometimes” to this item. In particular, affected *BRCA1*/*2* pathogenic variant carriers (7/20; 35%) as well as affected non-carriers (7/35; 20%) responded to this item with “often” or “sometimes.” As this item may measure concerns about private insurance enrollment, it seems reasonable for the MICRA-J to include questions about insurance.

A limitation of this study is that it was conducted at a single institution and involved a relatively small number of participants. The participants were categorized into five groups based on clinical characteristics, which introduced heterogeneity within the cohort. The small sample size of each group (i.e., < 10) makes it difficult to confirm internal consistency of each group, and the analyses of psychosocial effects in each group are subject to estimation uncertainty. Furthermore, as the main objective of this study was to confirm the reliability and validity of MICRA-J, psychological effects immediately after disclosure of the genetic test results or over the long term were not investigated. In actual clinical practice, changes in the MICRA-J scores should be examined to capture the psychosocial impact of genetic testing over time and how the psychosocial impact affects subsequent decision-making.

## Conclusions

The findings of this study suggest that the reliability and validity of the MICRA-J have been established. The MICRA-J, similar to the MICRA in other languages, is considered a useful tool to specifically measure the psychosocial impact of genetic testing.

## Supplementary Information

Below is the link to the electronic supplementary material.

Supplementary Material 1

## Data Availability

Data is provided within the manuscript or supplementary information files. The participants of this study did not provide written consent for their data to be shared publicly, hence, due to the sensitive nature of the research, the raw data are not available. To obtain the MICRA-J or permission for its use, please follow the appropriate procedures outlined at the FACIT.org website (https://www.facit.org/).
